# Supplementary material for: Controlling sharpness, SNR, and specific absorption rate for 3D fast‐spin echo at 7T by end‐to‐end learning
Source: Magn Reson Med. 2025 May 23;94(3):1026–43. doi: 10.1002/mrm.30533 (PMC12202734; doi:10.1002/mrm.30533)
Supplement: Supplementary file 1 — Figure S1. Exemplary transversal images of subject 1 obtained via the Pulseq 3D FSE sequence with T2w for standard, PSF‐ and SNR‐optimized VFA schemes (A–C, respectively). These images were subjected to a CNR analysis (ROIs to calculate the CNR of GM to WM and CSF to WM are depicted in rectangles). The nucleus caudatus was chosen as ROI for GM (orange), and ROIs for WM (blue) and CSF (orange) were selected in the vicinity. The resulting GM‐WM CNRs for standard VFA (green), the PSF‐optimization (blue) and SNR‐optimization (orange) are depicted in (B) and the corresponding CSF‐WM CNRs are depicted in (C). Figure S2. Exemplary transversal images of subject 2 obtained via the Pulseq 3D FSE sequence with T2w for standard, PSF‐, trade‐off‐ and SNR‐optimized VFA schemes (A–D, respectively). While this experiment reproduces the results from the other experiments (PSF‐optimized case shows better visibility of small structures, while SNR‐optimization yields significantly increased SNR), it should be noted that the trade‐off optimization preserves the benefits from the PSF‐optimization while providing significantly increased SNR. This is supported by quantitative analysis of the SNR (Figure S3) and the analysis of the PSF‐error (Figure S4). Figure S3. Quantitative analysis of the SNR via pseudo replica method for the standard, PSF‐, trade‐off‐ and SNR‐optimized case from the experiment shown in Figure S2. The mean value and the standard deviations within the ROIs (rectangle) are shown. It is worth noting that the trade‐off case surpasses the standard approach by approx. 7.2%. Figure S4. (A) The unencoded signal responses for standard, PSF‐, trade‐off‐ and SNR‐optimized VFA schemes for experiment shown in Figure S2. Note that the CSF contribution has been suppressed using an inversion pulse. The resulting invivo signal responses are in good agreement with those of white‐ and gray matter from the simulations (Figure 2). (B) The PSFs computed from the unencoded invivo signals (A) [file MRM-94-1026-s001.docx]

Supporting Material

**Controlling sharpness, SNR, and specific absorption rate for 3D fast‐spin echo at 7T by end‐to‐end learning**

Peter Dawood*^1,2^, Martin Blaimer^3^, Jürgen Herrler^4^, Patrick Liebig^4^, Simon Weinmüller^1^,

Shaihan Malik^5^, Peter M. Jakob^2^, Moritz Zaiss^1,6^

^1^Institute of Neuroradiology, University Hospital Erlangen, Friedrich-Alexander-Universität Erlangen-Nürnberg, Erlangen, Germany

^2^Experimental Physics 5, University of Würzburg, Würzburg, Germany

^3^Magnetic Resonance and X-ray Imaging Department, Fraunhofer Institute for Integrated Circuits IIS, Division Development Center X-Ray Technology, Würzburg, Germany

^4^Siemens Healthineers, Erlangen, Germany

^5^Department of Biomedical Engineering, King’s College, London, United Kingdom

^6^Department Artificial Intelligence in Biomedical Engineering, Friedrich-Alexander-Universität Erlangen-Nürnberg, Erlangen, Germany

**
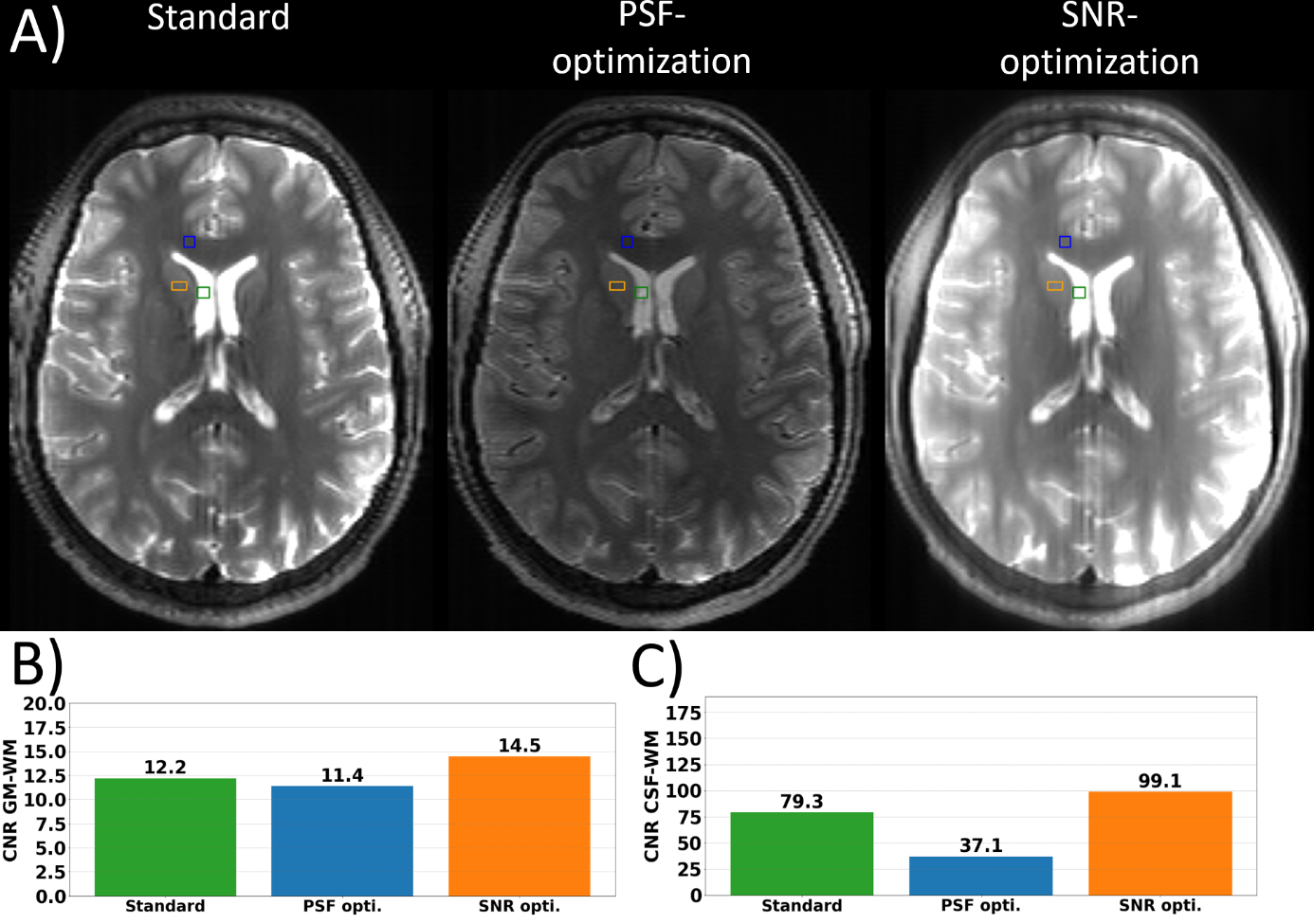
Figure S1**

Exemplary transversal images of subject 1 obtained via the Pulseq 3D FSE sequence with T2w for standard, PSF- and SNR-optimized VFA schemes (A-C, respectively). These images were subjected to a CNR analysis (ROIs to calculate the CNR of GM to WM and CSF to WM are depicted in rectangles). The nucleus caudatus was chosen as ROI for GM (orange), and ROIs for WM (blue) and CSF (orange) were selected in the vicinity. The resulting GM-WM CNRs for standard VFA (green), the PSF-optimization (blue) and SNR-optimization (orange) are depicted in B) and the corresponding CSF-WM CNRs are depicted in C).

**
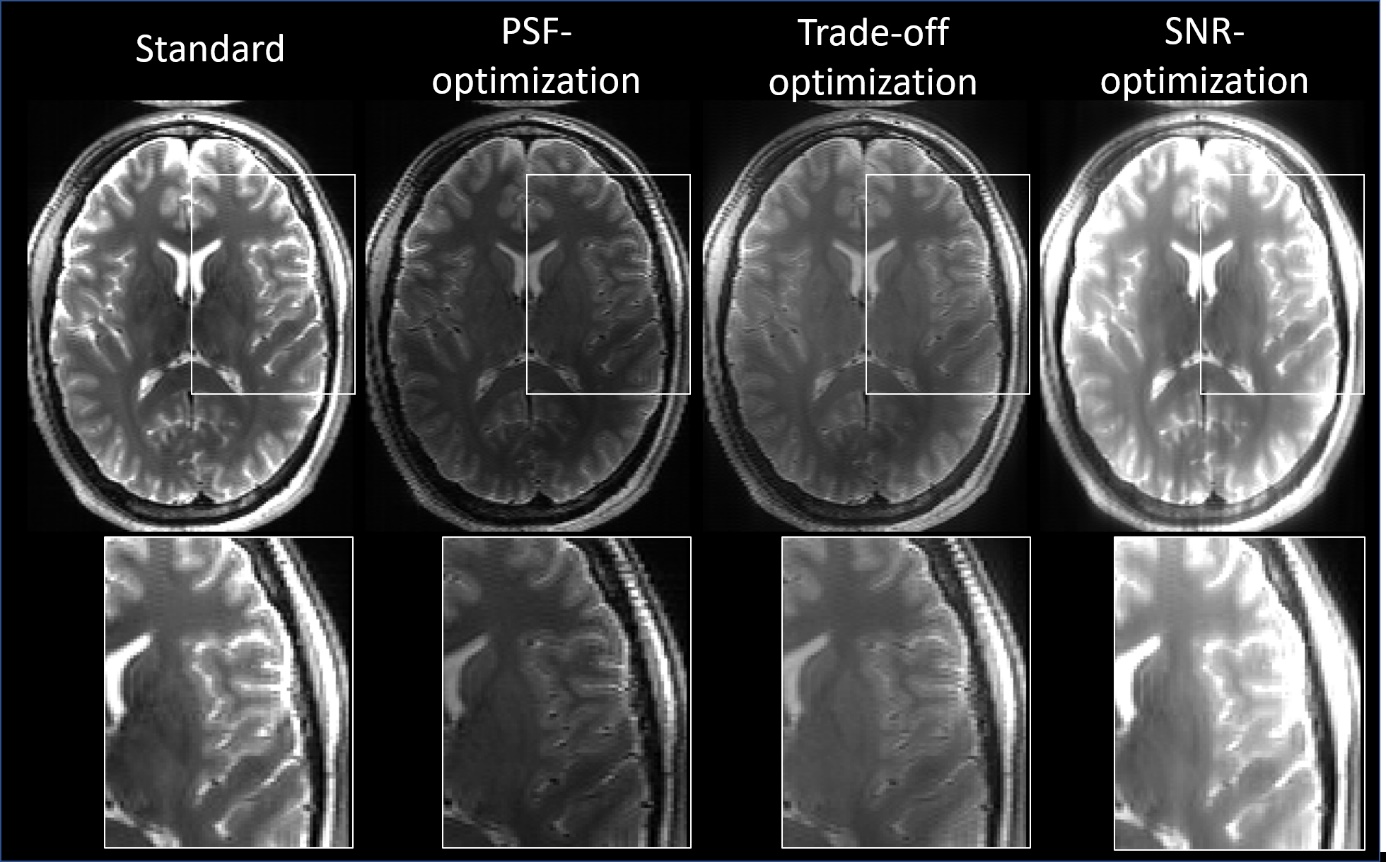
Figure S2**

Exemplary transversal images of subject 2 obtained via the Pulseq 3D FSE sequence with T2w for standard, PSF-, trade-off- and SNR-optimized VFA schemes (A-D, respectively). While this experiment reproduces the results from the other experiments (PSF-optimized case shows better visibility of small structures, while SNR-optimization yields significantly increased SNR), it should be noted that the trade-off optimization preserves the benefits from the PSF-optimization while providing significantly increased SNR. This is supported by quantitative analysis of the SNR (Figure S3) and the analysis of the PSF-error (Figure S4).


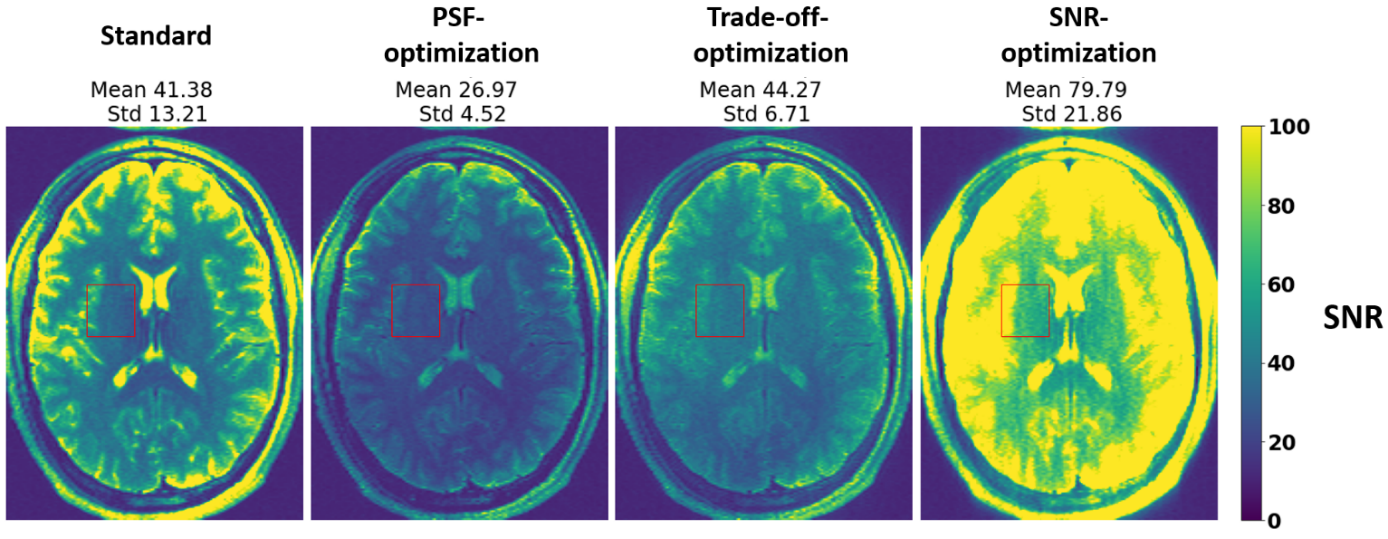


**Figure S3**

Quantitative analysis of the SNR via pseudo replica method for the standard, PSF-, trade-off- and SNR-optimized case from the experiment shown in Figure S2. The mean value and the standard deviations within the ROIs (rectangle) are shown. It is worth noting that the trade-off case surpasses the standard approach by approx. 7.2%.


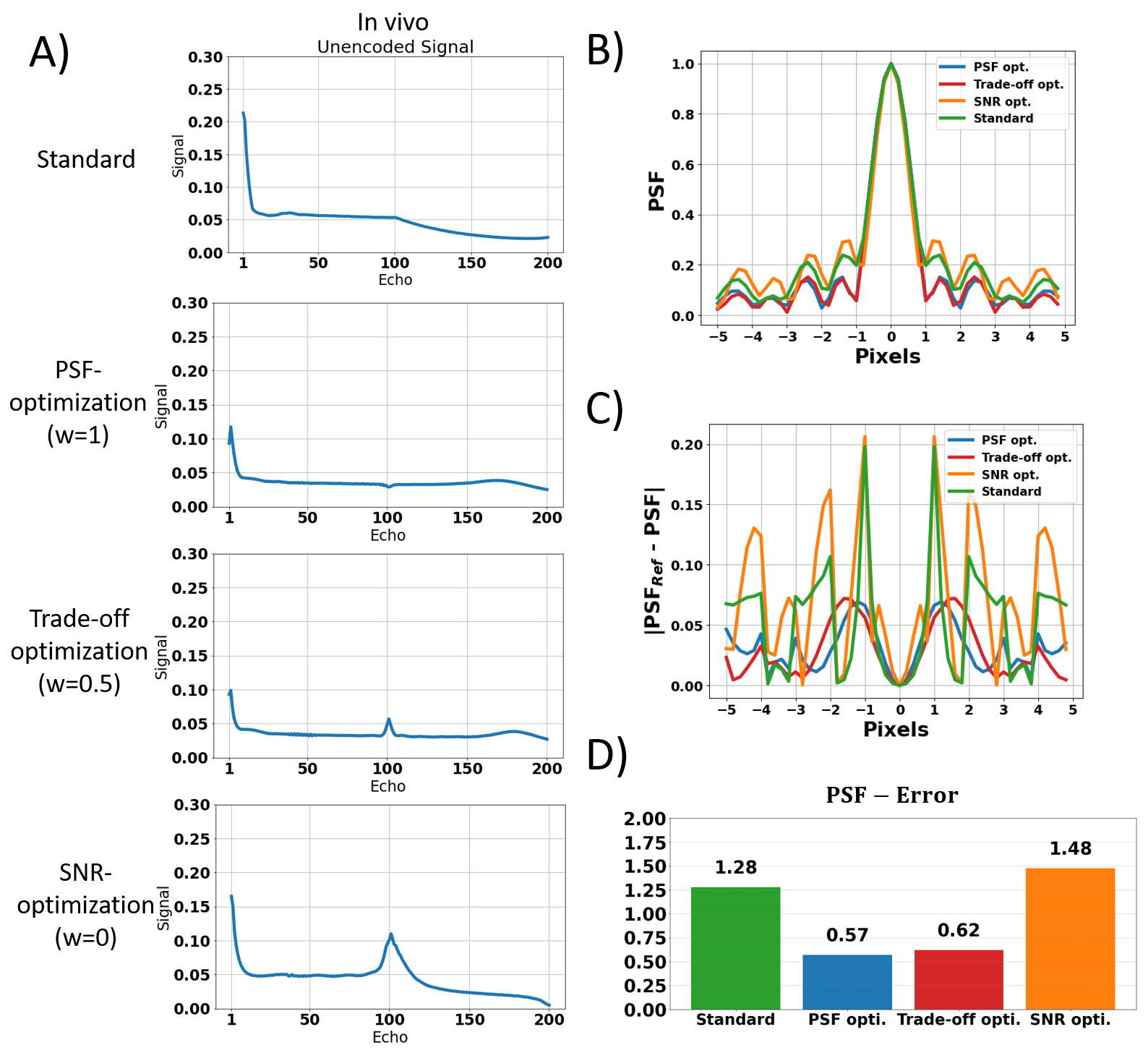


**Figure S4**

(A) The unencoded signal responses for standard, PSF-, trade-off- and SNR-optimized VFA schemes for experiment shown in Figure S2. Note that the CSF contribution has been suppressed using an inversion pulse. The resulting invivo signal responses are in good agreement with those of white- and gray matter from the simulations (Figure 2). (B) The PSFs computed from the unencoded invivo signals (A). (C) The differences of the invivo PSFs from a reference PSF (sinc), which corresponds to a uniform signal response over the echo train. (D) The quantitative error (i.e. the normalized L1-error to the ideal sinc-like reference PSF) of standard, PSF- and SNR-optimized VFA schemes. It is worth noting that the trade-off optimization yields a PSF-error similar to the PSF-optimization, supporting the impression of improved visibility of small structures in Figure S2. It is worth noting that the inversion RF pulse reduces the signal magnitudes of GM and WM compared to the case without the inversion RF pulse. However, the overall shape of the signal evolutions throughout the echo train remains preserved, resulting in PSFs that closely match those obtained in the absence of the inversion RF pulse.


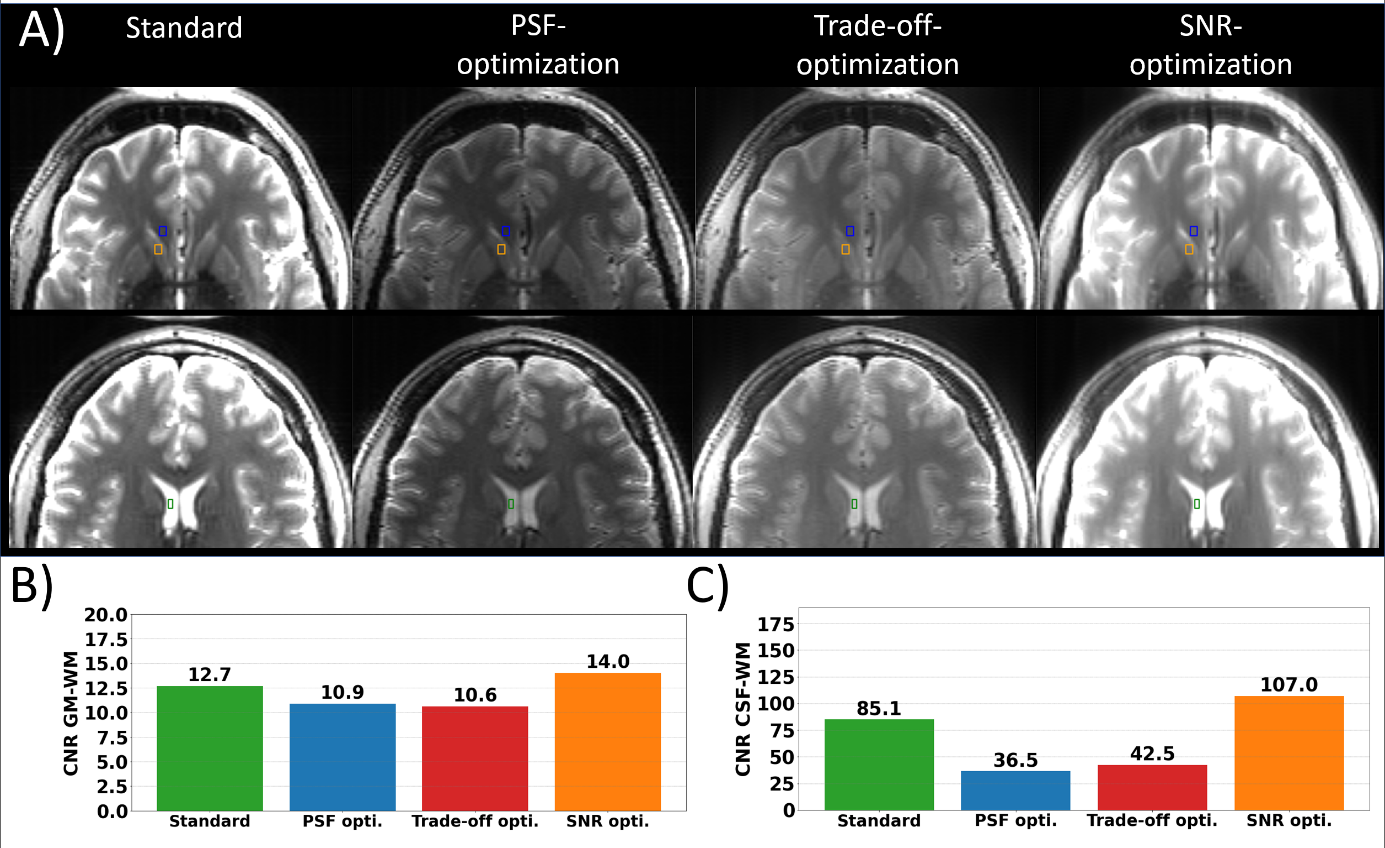


**Figure S5**

The testcase with trade-off optimization (Figure S2) was subjected to a CNR analysis. ROIs to calculate the CNR of GM to WM and CSF to WM are depicted in rectangles (A). The nucleus caudatus was chosen as ROI for GM (orange), and ROIs for WM (blue) and CSF (orange) were selected in the vicinity. The GM-WM CNR (B) is best for the SNR-optimized case (14.0, orange), while the trade-off optimization shows the worst CNR (10.6, red), which is slightly lower than for the PSF-optimization (10.9, blue). Standard yields a CNR of 12.7 (green). For the CSF-WM CNR (C), the SNR-optimized scheme achieves the highest CNR (107.0, orange), while the PSF-optimized scheme exhibits the lowest CNR (36.5, blue), illustrating again the trade-offs inherent in the different optimization strategies. Here, the trade-off optimization (red) again yields a comparable CNR to the PSF-optimization


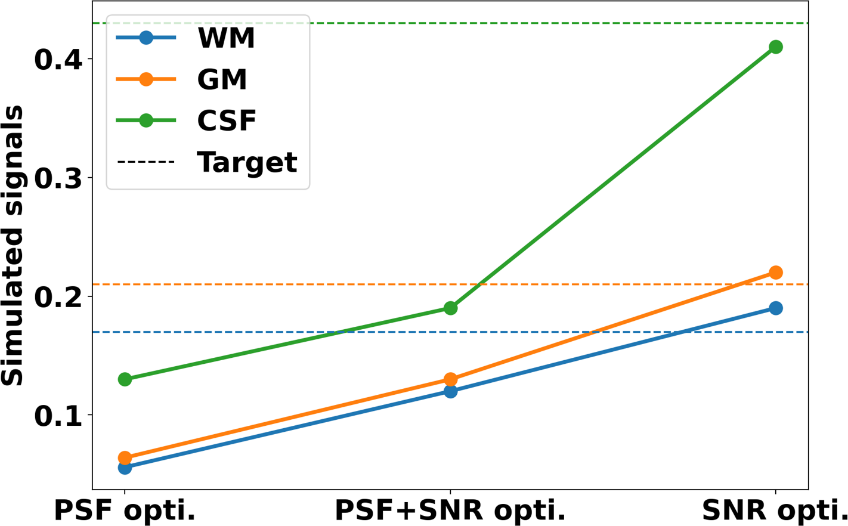


**Figure S6**

The relative signal levels for WM (blue), GM (orange) and CSF (green) in the simulated target spin echo image (dashed horizontal lines) in comparison to the signal levels in the simulated fast spin echo image for the PSF-optimization (left hand side), trade-off optimization (center) and SNR optimization (right hand side). As expected, the SNR-optimized case, where the L1 error to the spin echo target is minimized, shows the best agreement with the target contrast. This case matches the desired contrast relatively well. On the other hand, GM and WM signals progressively decrease as the L1 error weighting shifts towards the PSF error term.


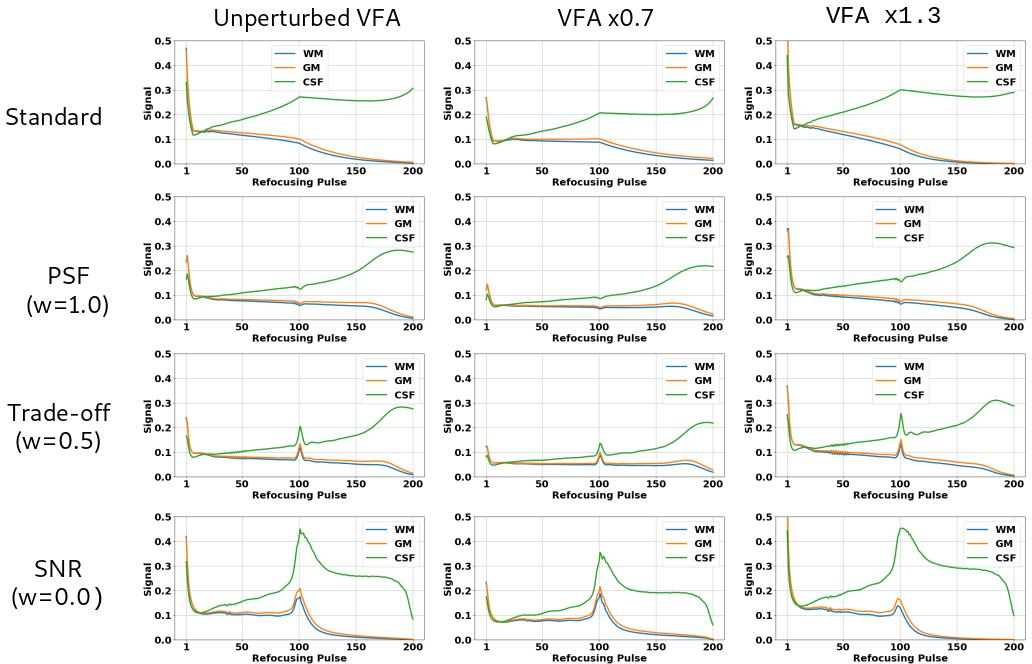
 **Figure S7**

For the simulation of the fast spin echo image, ideal B0 and B1+ maps were assumed. However, we tested different B1+ for the optimized trains by rescaling the variable flip angles (VFAs) and inspecting the corresponding signal responses. The singal responses are shown for the unperturbed VFAs (left hand side), and for VFAs scaled by 0.7 (center) and 1.3 (right hand side). As expected, the actual B1 level affects the signal intensities. However, the signal characteristics induced by the FA train are maintained. This is valid for both the vendor flip angle train and the optimized flip angle trains.
